# Supplementary figures and images for: Interspecific two-dimensional visual discrimination of faces in horses (Equus caballus)
Source: PLoS One. 2021 Feb 19;16(2):e0247310. doi: 10.1371/journal.pone.0247310 (PMC7894942; doi:10.1371/journal.pone.0247310)

1

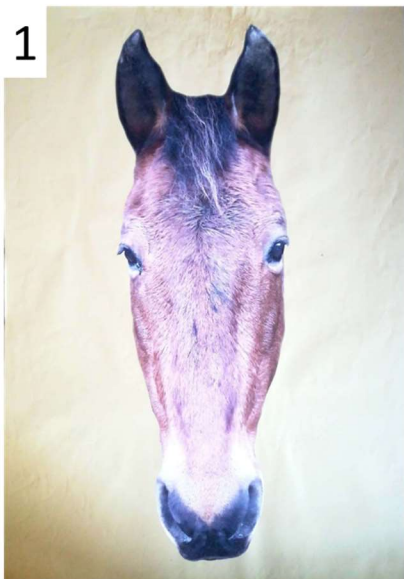

2

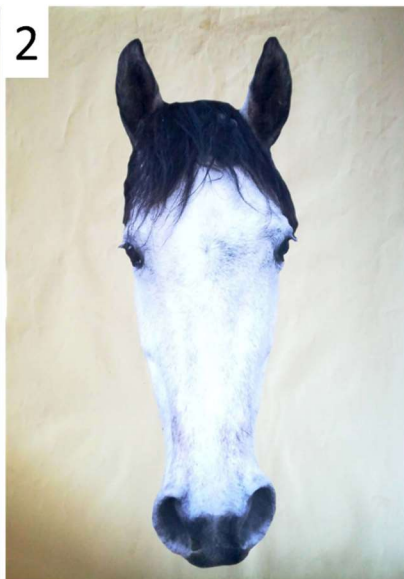

3

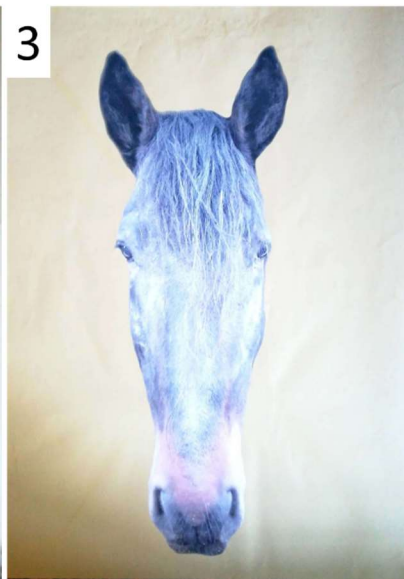

4

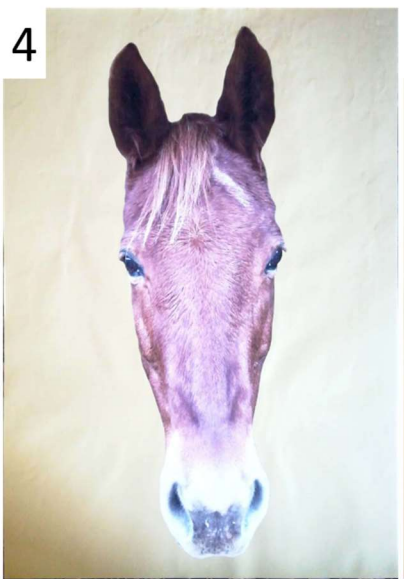

5

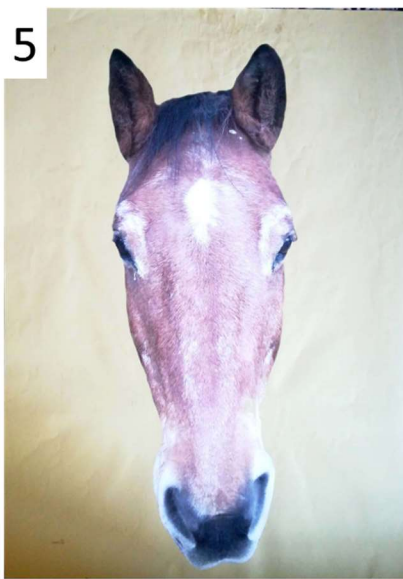

6

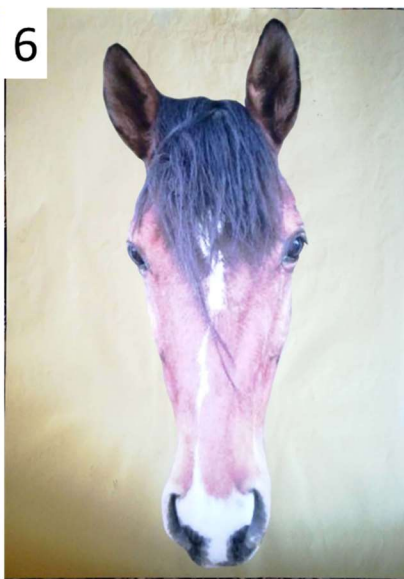

7

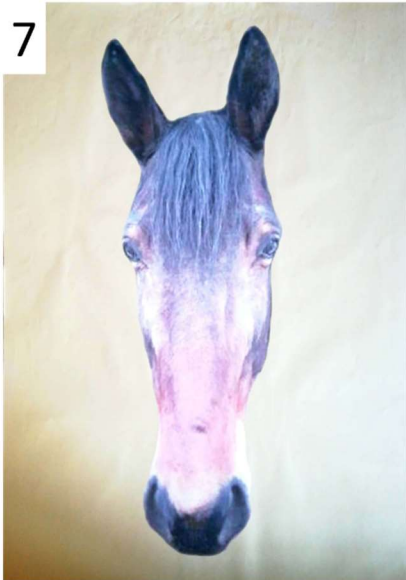

8

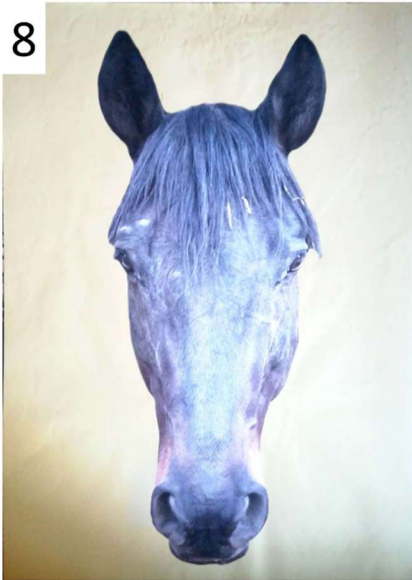

9

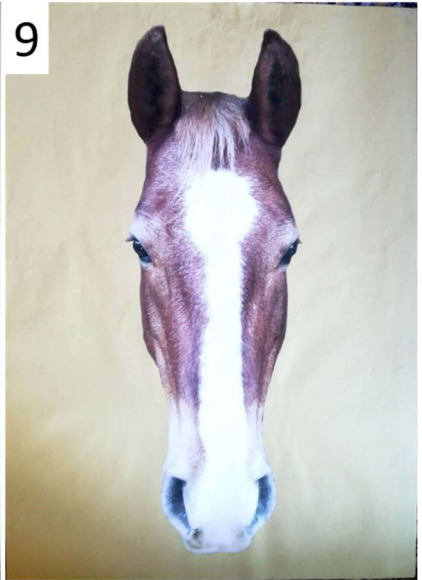

10

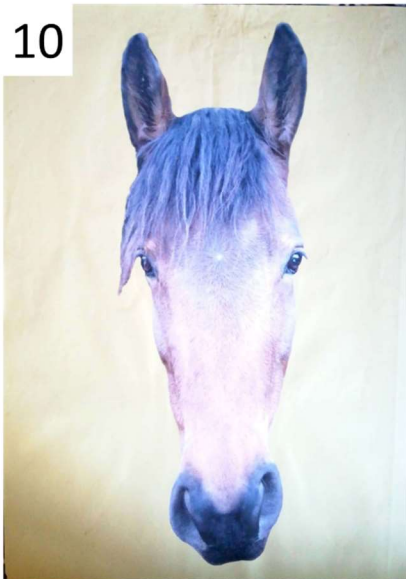

11

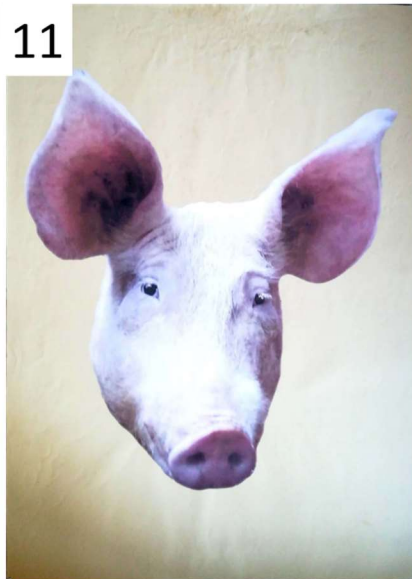

12

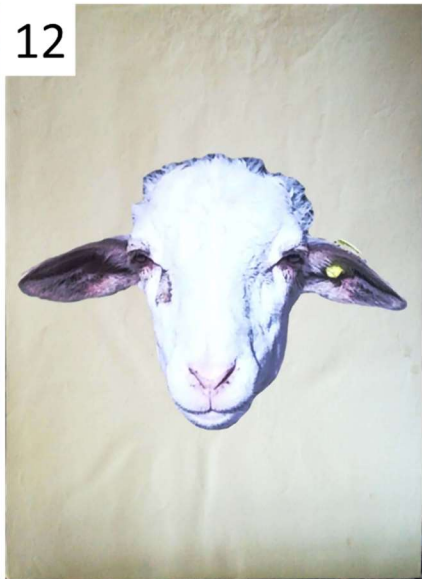

13

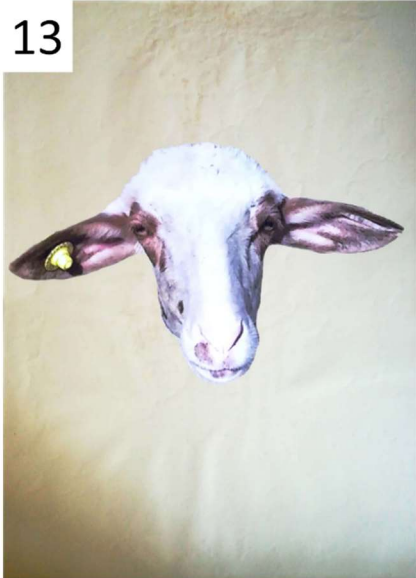

14

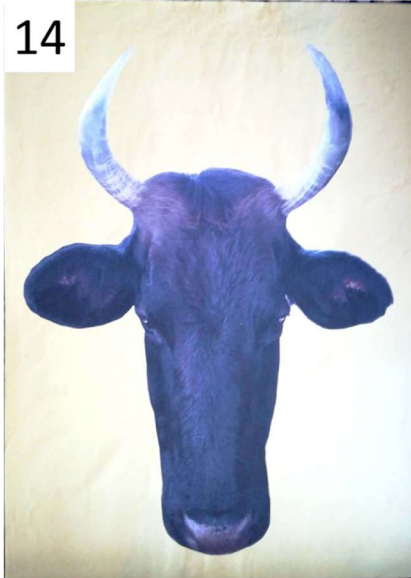

15

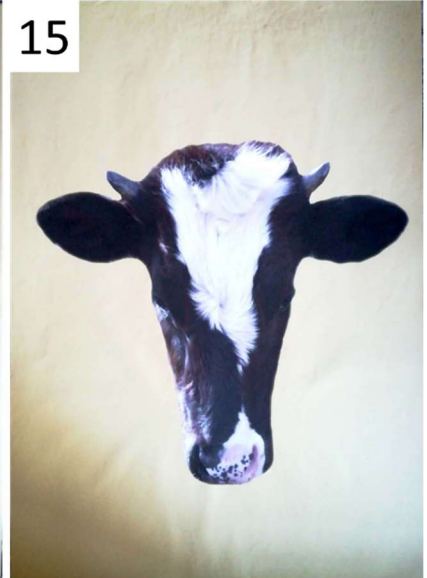

16

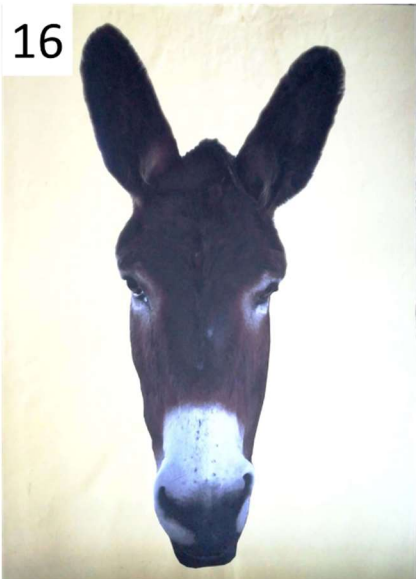

17

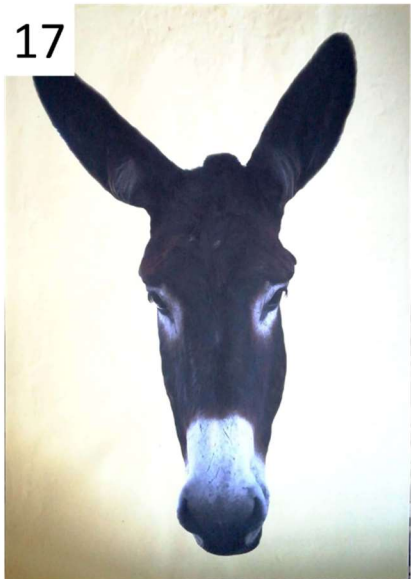

18

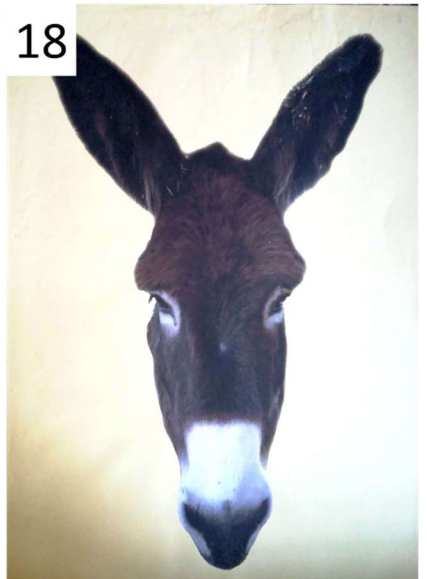

19

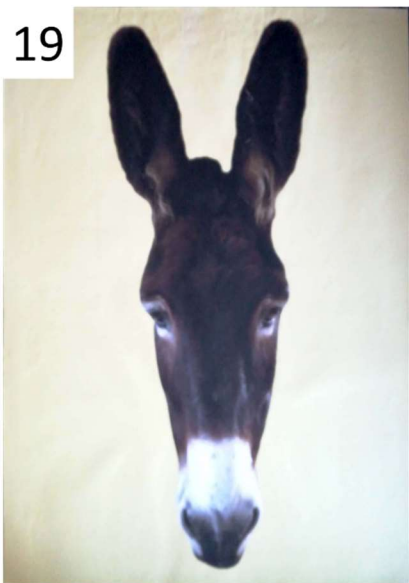

Scansato con CamScanner

20

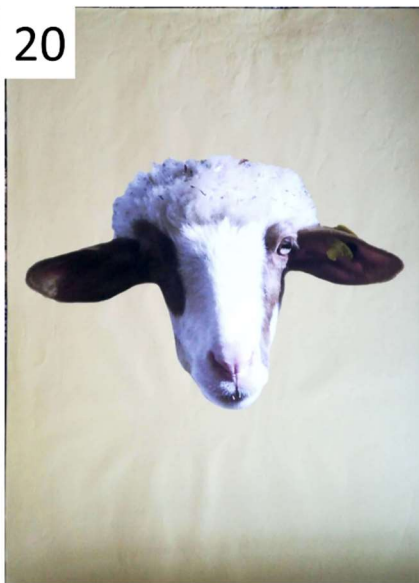

Scansato con CamScanner

Supplement: S1 Fig — (PDF) [file pone.0247310.s001.pdf]
