# Supplementary material for: Interspecific two-dimensional visual discrimination of faces in horses (Equus caballus)
Source: PLoS One. 2021 Feb 19;16(2):e0247310. doi: 10.1371/journal.pone.0247310 (PMC7894942; doi:10.1371/journal.pone.0247310)
Supplement: S1 Appendix — (PDF) [file pone.0247310.s002.pdf]

```
*Generalized Linear Mixed Models.
GENLINMIXED
  /DATA_STRUCTURE SUBJECTS=Horse*Session REPEATED_MEASURES=Trial
COVARIANCE_TYPE=DIAGONAL
  /FIELDS TARGET=Outcome TRIALS=NONE OFFSET=NONE
  /TARGET_OPTIONS DISTRIBUTION=BINOMIAL LINK=LOGIT
  /FIXED  EFFECTS=Session Horse_picture Other_picture USE_INTERCEPT=TRUE
  /RANDOM USE_INTERCEPT=TRUE SUBJECTS=Horse
COVARIANCE_TYPE=VARIANCE_COMPONENTS SOLUTION=FALSE
  /BUILD_OPTIONS TARGET_CATEGORY_ORDER=ASCENDING
INPUTS_CATEGORY_ORDER=ASCENDING MAX_ITERATIONS=100 CONFIDENCE_LEVEL=95
DF_METHOD=RESIDUAL COVB=MODEL PCONVERGE=0.000001(ABSOLUTE) SCORING=0
SINGULAR=0.000000000001
  /EMMEANS TABLES=Session COMPARE=Session CONTRAST=PAIRWISE
   /EMMEANS TABLES=Horse_picture COMPARE=Horse_picture CONTRAST=PAIRWISE
   /EMMEANS TABLES=Other_picture COMPARE=Other_picture CONTRAST=PAIRWISE
  /EMMEANS_OPTIONS SCALE=TRANSFORMED PADJUST=SEQSIDAK.
```

Generalized Linear Mixed Models

Notes

Output Created		10-NOV-2020 18:16:17
Comments		
Input	Active Dataset	DataSet1
	Filter	<none>
	Weight	<none>
	Split File	<none>
	N of Rows in Working Data File	270

Case Processing Summary

	N	Percent
Included	270	100,0%
Excluded	0	0,0%
Total	270	100,0%

Model Summary

Target		Outcome +/-
Probability Distribution		Binomial
Link Function		Logit
Information Criterion	Akaike Corrected	3552,604
	Bayesian	3590,035

Information criteria are based on the -2 log likelihood (3529,476) and are used to compare models. Models with smaller information criterion values fit better.

Data Structure[a]

	Subjects Session	Subjects Horse	Repeated Measures Trial	Target Outcome +/-
Data for First Subject	1	1	1	1
	1	1	2	0
	1	1	3	1
	1	1	4	0
	1	1	5	1
	1	1	6	0
	1	1	7	0
	1	1	8	1
	1	1	9	1
	1	1	10	0
Total Number of Levels	6	8	10	

a. Target: Outcome +/-

Classification
Overall Percent Correct = 72,6%[a]

Observed		Predicted 0	Predicted 1
0	Count	29	46
	% within Observed	38,7%	61,3%
1	Count	28	167
	% within Observed	14,4%	85,6%

a. Target: Outcome +/-

Fixed Effects[a]

Source	F	df1	df2	Sig.
Corrected Model	,969	23	246	,507
Session	,733	5	246	,600
Horsepicture	1,234	9	246	,274
Otherpicture1	1,030	9	246	,417

Probability distribution: Binomial
Link function: Logit[a]
a. Target: Outcome +/-

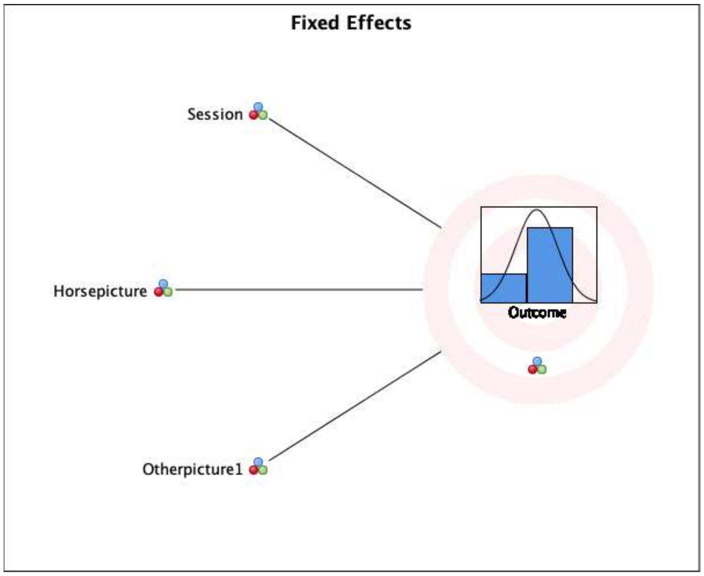

Overall Test Results

F	df1	df2	Sig.
1,030	9	246	,417
