# Supplementary material for: Interspecific two-dimensional visual discrimination of faces in horses (Equus caballus)
Source: PLoS One. 2021 Feb 19;16(2):e0247310. doi: 10.1371/journal.pone.0247310 (PMC7894942; doi:10.1371/journal.pone.0247310)
Supplement: S2 Appendix — (PDF) [file pone.0247310.s003.pdf]

```
*Generalized Linear Mixed Models.
GENLINMIXED
  /DATA_STRUCTURE SUBJECTS=Horse*Session REPEATED_MEASURES=Trial
COVARIANCE_TYPE=DIAGONAL
  /FIELDS TARGET=Time TRIALS=NONE OFFSET=NONE
  /TARGET_OPTIONS DISTRIBUTION=NORMAL LINK=IDENTITY
  /FIXED  EFFECTS=Session Horse_picture Other_picture USE_INTERCEPT=TRUE
  /RANDOM USE_INTERCEPT=TRUE SUBJECTS=Horse
COVARIANCE_TYPE=VARIANCE_COMPONENTS SOLUTION=FALSE
  /BUILD_OPTIONS TARGET_CATEGORY_ORDER=ASCENDING
INPUTS_CATEGORY_ORDER=ASCENDING MAX_ITERATIONS=100 CONFIDENCE_LEVEL=95
DF_METHOD=RESIDUAL COVB=MODEL PCONVERGE=0.000001(ABSOLUTE) SCORING=0
SINGULAR=0.000000000001
  /EMMEANS TABLES=Session COMPARE=Session CONTRAST=PAIRWISE
   /EMMEANS TABLES=Horse_picture COMPARE=Horse_picture CONTRAST=PAIRWISE
   /EMMEANS TABLES=Other_picture COMPARE=Other_picture CONTRAST=PAIRWISE
  /EMMEANS_OPTIONS SCALE=TRANSFORMED PADJUST=SEQSIDAK.
```

Generalized Linear Mixed Models

Notes

Output Created		10-NOV-2020 18:13:47
Comments		
Input	Active Dataset	DataSet1
	Filter	<none>
	Weight	<none>
	Split File	<none>
	N of Rows in Working Data File	270

Case Processing Summary

	N	Percent
Included	270	100,0%
Excluded	0	0,0%
Total	270	100,0%

Model Summary

Target		Time
Probability Distribution		Normal
Link Function		Identity
Information Criterion	Akaike Corrected	707,796
	Bayesian	745,226

Information criteria are based on the -2 log likelihood (684,668) and are used to compare models. Models with smaller information criterion values fit better.

Data Structure[a]

	Subjects Session	Subjects Horse	Repeated Measures Trial	Target Time
Data for First Subject	1	1	1	7,54
	1	1	2	7,98
	1	1	3	8,30
	1	1	4	8,28
	1	1	5	8,16
	1	1	6	8,00
	1	1	7	7,58
	1	1	8	7,92
	1	1	9	9,03
	1	1	10	8,33
Total Number of Levels	6	8	10	

a. Target: Time

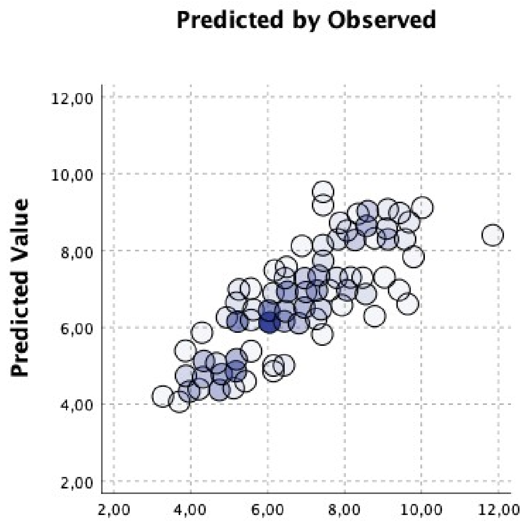

Fixed Effects[a]

Source	F	df1	df2	Sig.
Corrected Model	1,688	23	246	,028
Session	5,127	5	246	,000
Horsepicture	,975	9	246	,461
Otherpicture1	,641	9	246	,762

Probability distribution: Normal
Link function: Identity[a]
a. Target: Time

Fixed Effects

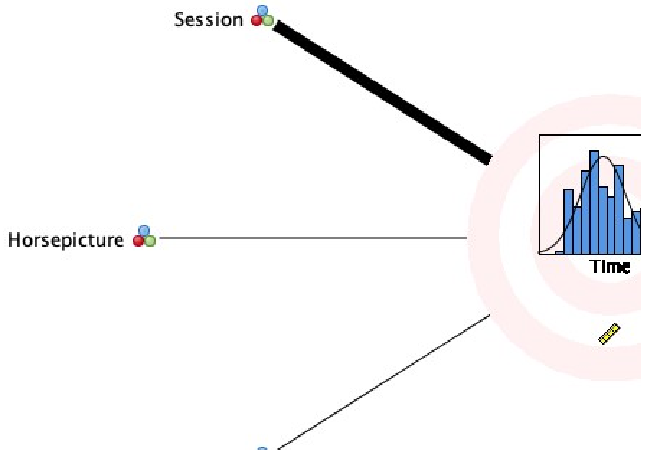

Overall Test Results

F	df1	df2	Sig.
,641	9	246	,762
