# Supplementary material for: Interspecific two-dimensional visual discrimination of faces in horses (Equus caballus)
Source: PLoS One. 2021 Feb 19;16(2):e0247310. doi: 10.1371/journal.pone.0247310 (PMC7894942; doi:10.1371/journal.pone.0247310)
Supplement: S3 Appendix — (PDF) [file pone.0247310.s004.pdf]

```
*Generalized Linear Mixed Models.
GENLINMIXED
  /DATA_STRUCTURE SUBJECTS=Horse*Session REPEATED_MEASURES=Trial
COVARIANCE_TYPE=DIAGONAL
  /FIELDS TARGET=Outcome TRIALS=NONE OFFSET=NONE
  /TARGET_OPTIONS DISTRIBUTION=BINOMIAL LINK=PROBIT
  /FIXED  EFFECTS=Session Horse picture Other picture
USE_INTERCEPT=TRUE
  /RANDOM USE_INTERCEPT=TRUE SUBJECTS=Horse
COVARIANCE_TYPE=VARIANCE_COMPONENTS SOLUTION=FALSE
  /BUILD_OPTIONS TARGET_CATEGORY_ORDER=ASCENDING
INPUTS_CATEGORY_ORDER=ASCENDING MAX_ITERATIONS=100 CONFIDENCE_LEVEL=95
DF_METHOD=RESIDUAL COVB=MODEL PCONVERGE=0.000001(ABSOLUTE) SCORING=0
SINGULAR=0.000000000001
  /EMMEANS TABLES=Session COMPARE=Session CONTRAST=PAIRWISE
   /EMMEANS TABLES=Horse picture COMPARE=Horse picture
CONTRAST=PAIRWISE
   /EMMEANS TABLES=Other picture COMPARE=Other picture
CONTRAST=PAIRWISE
  /EMMEANS_OPTIONS SCALE=ORIGINAL PADJUST=SEQSIDAK.
```

Generalized Linear Mixed Models

Notes

Output Created		08-NOV-2020 12:30:58
Comments		
Input	Active Dataset	DataSet1
	Filter	<none>
	Weight	<none>
	Split File	<none>
	N of Rows in Working Data File	270

Case Processing Summary

	N	Percent
Included	270	100,0%
Excluded	0	0,0%
Total	270	100,0%

Model Summary

Target		Outcome +/-
Probability Distribution		Binomial
Link Function		Probit
Information Criterion	Akaike Corrected	994,130
	Bayesian	1031,511

Information criteria are based on the -2 log likelihood (970,997) and are used to compare models. Models with smaller information criterion values fit better.

Data Structure[a]

	Subjects		Repeated Measures	Target
	Session	Horse	Trial	Outcome +/-
Data for First Subject	1	1	1	0
	1	1	2	1
	1	1	3	0
	1	1	4	0
	1	1	5	1
	1	1	6	1
	1	1	7	1
	1	1	8	1
	1	1	9	1
	1	1	10	0
Total Number of Levels	7	8	10	

a. Target: Outcome +/-

Classification
Overall Percent Correct = 75,9%[a]

Observed		Predicted 0	Predicted 1
0	Count	12	54
	% within Observed	18,2%	81,8%
1	Count	11	193
	% within Observed	5,4%	94,6%

a. Target: Outcome +/-

Fixed Effects[a]

Source	F	df1	df2	Sig.
Corrected Model	1,378	24	245	,117
Session	2,517	6	245	,022
Horsepicture	1,465	9	245	,162
Otherpicture1	,784	9	245	,631

Probability distribution: Binomial
Link function: Probit[a]
a. Target: Outcome +/-

Fixed Effects

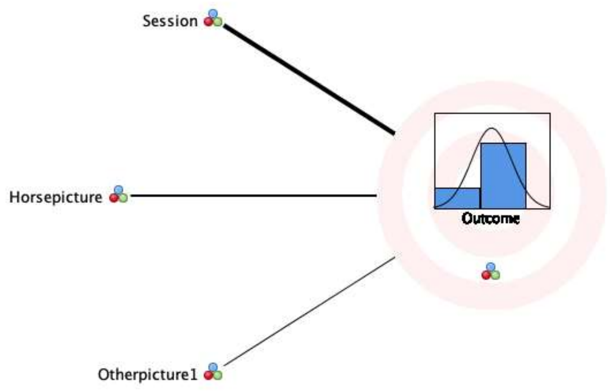

Overall Test Results

F	df1	df2	Sig.
,552	9	245	,836
