# Supplementary material for: Interspecific two-dimensional visual discrimination of faces in horses (Equus caballus)
Source: PLoS One. 2021 Feb 19;16(2):e0247310. doi: 10.1371/journal.pone.0247310 (PMC7894942; doi:10.1371/journal.pone.0247310)
Supplement: S4 Appendix — (PDF) [file pone.0247310.s005.pdf]

```
*Generalized Linear Mixed Models.
GENLINMIXED
  /DATA_STRUCTURE SUBJECTS=Horse*Session REPEATED_MEASURES=Trial
COVARIANCE_TYPE=DIAGONAL
  /FIELDS TARGET=Time TRIALS=NONE OFFSET=NONE
  /TARGET_OPTIONS DISTRIBUTION=NORMAL LINK=IDENTITY
  /FIXED  EFFECTS=Session Horse picture Other picture
USE_INTERCEPT=TRUE
  /RANDOM USE_INTERCEPT=TRUE SUBJECTS=Horse
COVARIANCE_TYPE=VARIANCE_COMPONENTS SOLUTION=FALSE
  /BUILD_OPTIONS TARGET_CATEGORY_ORDER=ASCENDING
INPUTS_CATEGORY_ORDER=ASCENDING MAX_ITERATIONS=100 CONFIDENCE_LEVEL=95
DF_METHOD=RESIDUAL COVB=MODEL PCONVERGE=0.000001(ABSOLUTE) SCORING=0
SINGULAR=0.000000000001
  /EMMEANS TABLES=Session COMPARE=Session CONTRAST=PAIRWISE
   /EMMEANS TABLES=Horse picture COMPARE=Horse picture
CONTRAST=PAIRWISE
   /EMMEANS TABLES=Other picture COMPARE=Other picture
CONTRAST=PAIRWISE
  /EMMEANS_OPTIONS SCALE=TRANSFORMED PADJUST=SEQSIDAK.
```

Generalized Linear Mixed Models

## Notes

| | | |
|---|---|---|
| Output Created | | 10-NOV-2020 18:20:19 |
| Comments | | |
| Input | Active Dataset | DataSet1 |
| | Filter | <none> |
| | Weight | <none> |
| | Split File | <none> |
| | N of Rows in Working Data File | 270 |

## Case Processing Summary

| | N | Percent |
|---|---|---|
| Included | 270 | 100,0% |
| Excluded | 0 | 0,0% |
| Total | 270 | 100,0% |

## Model Summary

| | | |
|---|---|---|
| Target | | Time |
| Probability Distribution | | Normal |
| Link Function | | Identity |
| Information Criterion | Akaike Corrected | 692,221 |
| | Bayesian | 729,602 |

Information criteria are based on the -2 log likelihood (669,088) and are used to compare models. Models with smaller information criterion values fit better.

## Data Structure[a]

| | Subjects | | Repeated Measures | Target |
|---|---|---|---|---|
| | Session | Horse | Trial | Time |
| Data for First Subject | 1 | 1 | 1 | 10,1600000000 |
| | 1 | 1 | 2 | 8,67000000000 |
| | 1 | 1 | 3 | 8,44000000000 |
| | 1 | 1 | 4 | 9,52000000000 |
| | 1 | 1 | 5 | 8,34000000000 |
| | 1 | 1 | 6 | 8,67000000000 |
| | 1 | 1 | 7 | 9,21000000000 |
| | 1 | 1 | 8 | 9,82000000000 |
| | 1 | 1 | 9 | 9,02000000000 |
| | 1 | 1 | 10 | 7,94000000000 |
| Total Number of Levels | 7 | 8 | 10 | |

a. Target: Time

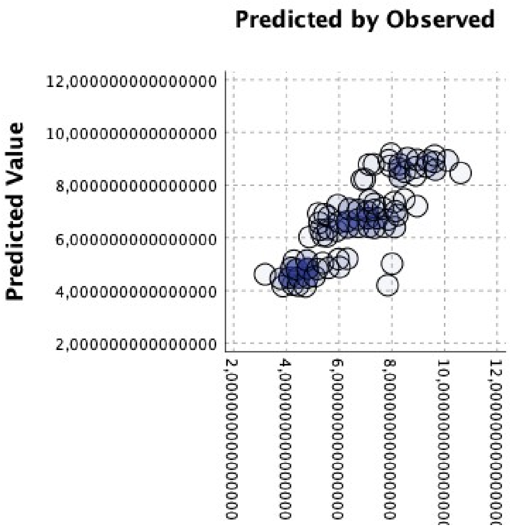


## Fixed Effects[a]

| Source | F | df1 | df2 | Sig. |
|---|---|---|---|---|
| Corrected Model | 2,130 | 24 | 245 | ,002 |
| Session | 6,887 | 6 | 245 | ,000 |
| Horsepicture | ,286 | 9 | 245 | ,978 |
| Otherpicture1 | ,575 | 9 | 245 | ,817 |

Probability distribution: Normal
Link function: Identity[a]

a. Target: Time

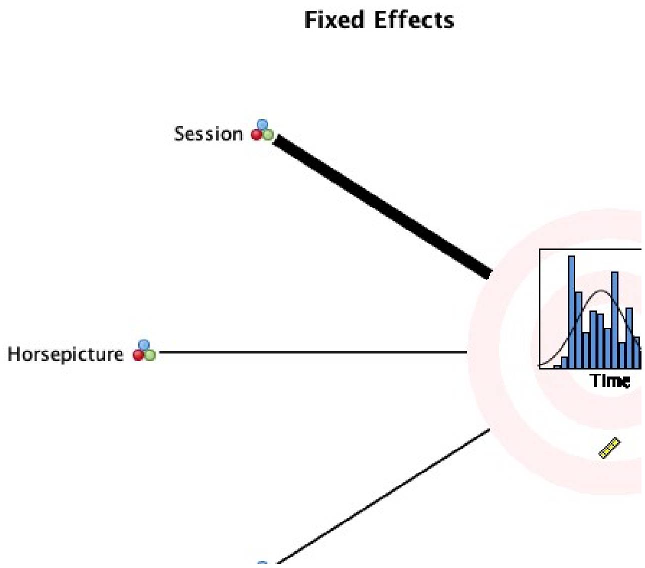


## Overall Test Results

| F | df1 | df2 | Sig. |
|---|---|---|---|
| ,575 | 9 | 245 | ,817 |
